# The Pathogenesis of Parkinson's Disease: A Complex Interplay Between Astrocytes, Microglia, and T Lymphocytes?

**DOI:** 10.3389/fneur.2021.666737

**Published:** 2021-05-26

**Authors:** Adina N. MacMahon Copas, Sarah F. McComish, Jean M. Fletcher, Maeve A. Caldwell

**Affiliations:** ^1^Department of Physiology, School of Medicine, Trinity College Dublin, Trinity Biomedical Sciences Institute, Dublin, Ireland; ^2^Trinity College Institute of Neuroscience, Trinity College Dublin, Dublin, Ireland; ^3^School of Biochemistry and Immunology, Trinity Biomedical Sciences Institute, Trinity College Dublin, Dublin, Ireland

**Keywords:** Parkinson's disease, astrocyte, microglia, T lymphocytes, Th17 cell, neuroinflammation

## Abstract

Parkinson's disease (PD), the second most common neurodegenerative disease, is characterised by the motor symptoms of bradykinesia, rigidity and resting tremor and non-motor symptoms of sleep disturbances, constipation, and depression. Pathological hallmarks include neuroinflammation, degeneration of dopaminergic neurons in the substantia nigra pars compacta, and accumulation of misfolded α-synuclein proteins as intra-cytoplasmic Lewy bodies and neurites. Microglia and astrocytes are essential to maintaining homeostasis within the central nervous system (CNS), including providing protection through the process of gliosis. However, dysregulation of glial cells results in disruption of homeostasis leading to a chronic pro-inflammatory, deleterious environment, implicated in numerous CNS diseases. Recent evidence has demonstrated a role for peripheral immune cells, in particular T lymphocytes in the pathogenesis of PD. These cells infiltrate the CNS, and accumulate in the substantia nigra, where they secrete pro-inflammatory cytokines, stimulate surrounding immune cells, and induce dopaminergic neuronal cell death. Indeed, a greater understanding of the integrated network of communication that exists between glial cells and peripheral immune cells may increase our understanding of disease pathogenesis and hence provide novel therapeutic approaches.

## Introduction

Parkinson's disease (PD) is the second most common neurodegenerative disease affecting 1–2% of the population over the age of 65 (1) and it is estimated that the number of cases will exceed 12 million individuals in 2040 (2). PD is characterised by the degeneration of dopamine neurons in the substantia nigra (SN) of the midbrain, with concomitant loss of their axons that project to the striatum along the nigrostriatal pathway. This results in loss of the neurotransmitter dopamine which leads to the primary motor symptoms of PD, which were first described by James Parkinson in 1817 as a heterogeneous manifestation (3). These include bradykinesia, ataxia, tremor, rigidity, and postural instability which present themselves clinically once the levels of striatal dopamine decrease by 70% (4). Another key pathological feature of PD is the presence of protein inclusions known as Lewy bodies (5). The protein α-synuclein (α-syn) is a major component of Lewy bodies (6) and its mutant forms can cause familial PD (7–10).

In the central nervous system (CNS), the continuous interactions of neurons, glia, and the microenvironment are key for the maintenance of neural homeostasis and failures in this homeostatic state leads to neurodegenerative conditions such as PD. In recent years the role of inflammatory processes in the death of dopamine neurons has come to the fore and is now considered vital to this process (11–13). Gliosis is a typical pathological feature of many neurodegenerative diseases and emerging evidence indicates that sustained activation of microglia and astrocytes is central to dopaminergic degeneration in PD (14). Indeed, activation of microglia in people with PD was described for the first time in 1988 (15), and this phenomenon has also been described in animal models (16, 17). Wilson et al. have recently described a PET ligand ^11^C-BU99088 which is expressed on reactive astroglia, and as such, was reflective of astroglial pathology in people with PD and demonstrated a role for astroglia in the initiation and progression of PD (18). Astrocyte reactivity was also detected in the SN of patients with this condition [for review see (19)] and post-mortem studies of nigral tissue homogenates revealed multiple alterations in biochemical parameters attributed to astrocyte dysfunction including a global reduction of glutathione levels, mitochondrial damage, and accumulation of extracellular toxins (20). In addition, clinical and basic research have revealed a role for both the innate and adaptive immune system in PD [for review see (21, 22)]. In fact, the available data suggests that in PD there is a response not only by glial cells but also by peripheral immune cells, suggesting that an interplay between these cell types contributes to pathophysiology. We will review the role of astrocytes and microglia in PD, taking into account the emerging role of peripheral immune cells and its implications for disease pathogenesis.

## Astrocytes and Their Role in Parkinson's Disease

Astrocytes are the most abundant non-neuronal cell type in the vertebrate nervous system (23) with the critical task of maintaining structural support and homeostasis. Their roles include provision of metabolic support, encapsulation of neuronal synapses (24), promotion of synaptogenesis (25), and control of the permeability of the blood brain barrier (BBB) (26). Classically they are recognised for their ability to secrete a number of neurotrophic factors such as glial derived neurotrophic factor (GDNF) and mesencephalic astrocyte derived neurotrophic factor (MANF); both of which have been shown to offer a degree of neuroprotection to dopamine neurons both *in vitro* and *in vivo* (27–29). However, in addition to this there is a growing appreciation of the role astrocytes play in neuroinflammation (30) in many neurodegenerative conditions including PD. Recently it has been shown that astrocytes become reactive in response to activated microglial secreted signals such as IL-1α, TNF-α, and C1q and as such adopt a pro-inflammatory phenotype (31, 32). Indeed, this astrocyte phenotype has been shown to exist in the post-mortem brain tissue of people with PD (31). Furthermore, astrocytes can also adopt a pro-inflammatory phenotype by endocytosis of α-syn released by neurons; they secrete cytokines, IL-1α, IL-1β, and IL-6 which are correlated to α-syn load (33). Moreover, α-syn accumulation in human astrocytes *in vitro* resulted in severe cellular stress which included mitochondrial, lysosomal, and endoplasmic reticulum deficiencies (34, 35). Indeed, these astrocytes responded to this stress by sending out nanotubes, which behaved like tunnels, and enabled the transfer of intracellular α-syn inclusions to nearby cells, indicating that astrocytes are critically important in the pathogenesis of PD (35). Interestingly, Yun et al., demonstrated that NLY101, a GLP-1R agonist is neuroprotective in the pre-formed α-syn fibril model of PD. It acts to prevent astrocyte stimulation by activated microglia and in so doing protects dopamine neurons and prevents behavioural deficits (36). Taken together, these studies would suggest that astrocyte dysfunction is a very strong contributor to the pathogenesis of PD.

### Impact of Parkinson's Disease Related Genetics on Astrocyte Function

There are monogenic mutations identified in 20 genes that have been implicated in the pathogenesis of PD (37). Interestingly, a study by Zhang et al. (38) compared the transcriptome of human astrocytes to neurons, and found upregulation of some of these monogenic mutations in astrocytes was to a similar level and sometimes higher than that of neurons [for review see (39)]. This would strongly support the potential contribution of astrocytes to the pathogenesis of these familial forms of PD. Altered levels of these genes lead to many changes in astrocyte function including impaired glutamate uptake, liposomal homeostasis, lysosomal, and mitochondrial dysfunction and inflammatory response (Table 1).

**Table 1:** The role of genes that are causative in Parkinson's disease pathogenesis and their implications in astrocytes.

**Gene/Protein**	**Model used**	**Main findings**	**References**
*Park 7*/DJ-1	DJ-1 KO mice	• Alteration in cholesterol levels • Alterations in membrane fluidity and in lipid raft dependent endocytosis • Altered glutamate uptake capacity	(40)
*Park 7*/DJ-1	DJ-1 KO mice	• Regulates anti-inflammatory role of astrocytes through Prostaglandin D2 synthase expression	(41)
*PARK2*/Parkin	Parkin KO mice	• KO astrocytes exhibit exaggerated ER stress, JNK activation cytokine release and reduced neurotrophic factors	(42)
*PINK1*/PINK1	PINK1 KO mice	• Reduced astrocyte differentiation, increased p38 activation	(43)
*PINK1*/PINK1	PINK1 KO mice astrocytes	• Increased levels of iNOS, NO, TNF-α, and IL-1β	(44)
*PINK1*/PINK1	PINK1 KO rat pups	• PINK1 phosphorylation of ubiquitin is predominately in astrocytes	(45)
*SNCA/*α-syn	Human *SNCA* over-expressing primary foetal astrocytes	• α-syn changed expression of GF production and secretion, e.g., EGF, PDGF, VEGF, and their receptors • α-syn changed expression of IGF related proteins	(46)
*SNCA*/α-syn	A53T WT α-syn in mouse astrocytes	• Disrupted glutamate uptake • Increased neuronal cell death by overexpressing astrocytes	(47)
*LRRK2*/LRRK2	*G2019S- LRRK2*-Tg het mice astrocytes + α-syn	• Increased ER stress proteins • Increased cell death with α-syn • Mitochondrial dysfunction with α-syn	(48)
*GBA1*/GCase	Mice *GBA1 D409V* knockin astrocytes	• Defects in lysosomes • Defects in TLR4-dependent cytokine release	(49)
*LRRK2*/LRRK2	Human iPSC *LRRK2-G2019S*- astrocytes and neurons	• Impaired autophagy in astrocytes • PD astrocytes accumulate and transfer α-syn to healthy dopamine neurons	(50)
*LRRK2*/LRRK2	Human iPSC *LRRK2-G2019S*—MB astrocytes	• Downregulation of MMP2 and TGFβ	(51)
*LRRK2*/LRRK2	Human iPSC *LRRK2-G2019S*—astrocytes one patient also with *GBA N370S*	• Increased expression of α-syn, • Altered metabolism• Disrupted Ca^2+^ homeostasis• Increased cytokine release following inflammatory stimulation	(52)
*GBA1*/GCase	Human iPSC-derived astrocytes from GD1 with genotype *N370S/N370S* or *N370S/c.84insG*	• Abnormal α-syn accumulation due to Impaired lysosomal cathepsin activity • Increased inflammatory response	(53)

*LRRK2, leucine rich repeat kinase 2; GCase, β-Glucocerebrosidase; α-syn, alpha-synuclein; iPSC, pluripotent stem cells; MMP2, matrix metalloproteinase 2; PINK1, PTEN-induced putative kinase-1; iNOS, inducible nitric oxide synthase; MB, midbrain; GF, growth factor; EGF, epidermal growth factor; PDGF, platelet derived growth factor; VEGF, vascular endothelial growth factor; IGF, Insulin like growth factor; GD, Gaucher disease*.

#### DJ-1/PARK7

Some of these genes have been studied to a greater extent than others with respect to their roles in astrocyte biology. DJ-1, encoded by the *PARK7* gene, causes early onset autosomal recessive PD (54) and is probably the most extensively studied. Mullett and Hinkle utilised neuron-astrocyte co-cultures to demonstrate that siRNA knockdown of DJ-1 in mouse astrocytes impairs their ability to protect against neurotoxins such as rotenone relative to wild type control astrocytes (55). In addition, studies using DJ-1 knockout mice astrocytes from postnatal day 1 cerebral cortices have also shown that loss of this gene can cause alterations in cholesterol levels and glutamate uptake via regulation of the expression of flotillin-1 and caveolin-1 (40). Furthermore, Choi et al. demonstrated using astrocytes from DJ-1 knockout mice that its deficiency in astrocytes reduced expression of prostaglandin D2 synthase and subsequent secretion of prostaglandin D2 demonstrating that DJ-1 is involved in the regulation of the anti-inflammatory role of astrocytes through prostaglandin D2 synthase expression (41). Moreover, a study from the Kahle laboratory found that DJ-1 knockout mice astrocytes produced 10 times more nitric oxide (NO) than littermate controls when treated with lipopolysaccharide (LPS), a TLR4 agonist, and interestingly lentiviral reintroduction of DJ-1 restored the response to LPS (56). Taken together, these studies demonstrate that DJ-1 is an important regulator of the pro-inflammatory response and that its knockout in astrocytes deregulates inflammatory associated damage.

#### PARK2 and PINK1

Both *PARK2* and *PINK1* are expressed at similar levels in both astrocytes and neurons (38, 39, 57). Interestingly, astrocytes deficient in Parkin, encoded by the *PARK2* gene, demonstrated a stress induced increase in NOD2 expression; a receptor which integrates ER stress and inflammation and these astrocytes exhibited increased cytokine release and decreased secretion of neurotrophic factors (42). Parkin has also been shown to be involved in the response of astrocytes to an inflammatory signal; activation with TNF-α results in Parkin upregulation whereas activation with IL-1β results in Parkin downregulation (58). *PINK1* expression, which encodes the protein PTEN-induced putative kinase 1 (PINK1), is a loss of function mutation that is associated with early onset PD (59). Its expression increases during embryonic development and it was also shown to affect the development of GFAP-positive astrocytes (43). Indeed when PINK1 is knocked out in astrocytes, this causes them to exhibit reduced differentiation (43), reduced neurotrophic factor release, endoplasmic reticulum (ER) stress, JNK activation (42), and generate elevated levels of iNOS, NO, TNF-α, and IL-1β via NF-κB signalling (44). Interestingly, the steady state levels of PINK1 protein are very low even in cells that express PINK1, because PINK1 is normally targeted for degradation after mitochondrial import by a process that is dependent on mitochondrial membrane potential (60). While the high penetrance of PINK1 mutations establish its critical function for maintaining neurons the activity of PINK1 in primary neurons has been difficult to detect. However, Barodia et al. determined the levels of PINK1 in neurons, astrocytes, microglia, and oligodendrocyte progenitor cells (OPCs) cultured from wild type and PINK1 knockout rat pups and showed that PINK1-dependent ubiquitin phosphorylation is predominately in astrocytes suggesting that the contribution of astrocyte dysfunction to PD pathogenesis warrants further investigation (45).

#### SNCA

*SNCA* encodes for the protein α-syn and missense mutations as well as duplication or triplication of this gene have been shown to lead to the development of rare hereditary forms of PD [for review see (61)]. Interestingly, one such mutation of the *SNCA* gene is also associated with an increase in α-syn aggregation in astrocytes; Gu et al. demonstrated that when *A53T* α-syn was selectively expressed in murine astrocytes it led to disrupted glutamate uptake and increased neuronal death (47). Indeed, *SNCA* is one of the PD associated genes that is expressed at higher levels in neurons than in astrocytes (38, 57). In 1997, Spillantini et al. reported that α-syn was the major component of Lewy bodies in the brains of people with idiopathic PD (6) and inclusions of α-syn have been found in astrocytes as well as neurons in post-mortem brains of people with PD (62, 63). Interestingly, it has been shown that neuronal α-syn can be directly transferred to astrocytes through sequential exocytosis and endocytosis (33, 64). In addition, overexpression of human α-syn in human primary foetal astrocytes resulted in significant changes in the profile of growth factor expression and release. The most remarkable changes were in epidermal growth factor (EGF), platelet derived growth factor (PDGF), vascular endothelial growth factor (VEGF), and their receptors as well as in insulin like growth factor (IGF) related proteins (46). Further analysis, using bioinformatics, revealed possible interactions between α-syn and EGFR and GDNF (46).

#### LRRK2 and GBA

*LRRK2* encodes the protein leucine-rich repeat kinase 2 (LRRK2) and is causative for a dominantly inherited form of PD (65). Mutations in *LRRK2* have been recognised as genetic risk factors for both familial and idiopathic forms of PD (66). When the pathogenic mutation in *LRRK2, LRRK2-G2019S*, was expressed in mice it resulted in increased ER stress and cell death in astrocytes that was exacerbated by the addition of α-syn (48). *GBA* encodes an enzyme important in glycolipid metabolism, beta-glucocerebrosidase (GCase). The effects of a mutation in GCase encoded by the *GBA1* gene in astrocytes has been studied in mice with knockin of *GBA D490V*. This resulted in defects in lysosomes and in TLR4-dependent release of cytokines (49).

It is noteworthy that the vast majority of the studies carried out to date on the possible implications of the expression of PD related genes in astrocytes have been conducted in rodents. However, in the last 2 years a small number of studies have begun to emerge in the literature where human induced pluripotent stem cells (iPSC) carrying PD related mutations have been differentiated into astrocytes. It is not surprising that the majority of these are studying the *LRRK2-G2019S* mutation as this is the most common PD related mutation (67). A study by di Domenico et al. has shown that there is impaired autophagy in astrocytes with a *LRRK2-G2019S* mutation and that they can accumulate and transfer α-syn to healthy dopamine neurons (50). Another study patterned regional midbrain astrocytes from iPSC containing familial *LRRK2-G2019S* mutation and from healthy controls. RNAseq analysis revealed downregulation of genes such as *TGF*β*1* and *MMP2* (51). TGFβ1 has previously been shown to inhibit the inflammatory microglial response in a rat model of PD (68), and MMP2 has been shown to be capable of degrading aggregates of α-syn (69). This suggests that these PD astrocytes have a reduced neuroprotective capacity and so may contribute to pathology (51). Further evidence for an increased expression of α-syn in astrocytes has been provided by Sonninen et al. using astrocytes differentiated from iPSC also with *LRRK2-G2019S* mutation. These astrocytes exhibited altered metabolism and increased cytokine release following inflammatory stimulation (52). iPSC-derived astrocytes with two different *GBA1* mutations (*N370S/N370S* and *N370S/c.84insG*) have also demonstrated abnormal α-syn accumulation due to impaired lysosomal cathepsin activity and had an enhanced inflammatory response (53). Taken together these studies are consistent with the role for both *LRRK2* and *GBA1* mutations in accumulation of α-syn and an increased inflammatory response in astrocytes as contributors to PD pathology.

#### Other Parkinson's Disease Risk Genes

In addition to the six PD risk genes mentioned above there are a number of other risk genes in which there is very high confidence that they are actual PD genes; these are *PLA2G6, ATP13A2, FBXO7*, and *VPS35* [for review see (37)]. Interestingly, the levels of *PLA2G6, ATP13A2*, and *FBXO7* gene expression has been shown to be the same in neurons as in astrocytes but that of *VPS35* is greater in astrocytes than in neurons (38, 39, 57). *PLA2G6* encodes Ca^2+^-independent phospholipase A2 (iPLA2), an enzyme responsible for catalysing the release of fatty acids from phospholipids. A recent study by Strokin and Reiser in 2017 demonstrated that astrocytes from mice with a mutation in the *Pla2g6* gene, that were treated with Ru360 (a blocker of mitochondrial Ca^2+^ uniporter), or with rotenone, had a reduced rate of glutamate-induced Ca^2+^ influx, which was ~2-fold lower than in wild type controls (70). The *ATP13A2* gene encodes a transmembrane lysosomal P5-type ATPase and its missense or truncation mutation leads to lysosomal dysfunction (71). A study using primary astrocytes from the mouse midbrain with a deficiency in *ATP13A2* demonstrated intense inflammation, which exacerbated dopamine neuron damage following MPP^+^ exposure (71). Furthermore, this same study showed that astrocytes lacking *ATP13A2* had increased lysosomal membrane permeabilisation and cathepsin B release, which in turn led to the activation of the NLRP3 inflammasome. This led to increased production of IL-1β and suggested that there is a direct link between the astrocyte lysosome and neuroinflammation in PD (71). Lysosomal degradation was also shown using human iPSC, from healthy controls or from patients carrying a mutation in lysosomal *ATP13A2*, which were differentiated into midbrain dopamine neurons and astrocytes (72). This study showed that astrocytes rapidly internalised α-syn and when they were co-cultured with neurons, this led to a decreased accumulation of α-syn in neurons and as a consequence diminished interneuronal transfer of α-syn. Interestingly, loss of this protective function of astrocytes was seen with *ATP13A2* deficiency, suggesting that this gene function in astrocytes, contributes partially to PD pathology (72).

Mutations in the *FBXO7* (*PARK15*) gene have been implicated in a juvenile form of PD. When this gene was deleted in tyrosine hydroxylase neurons this resulted in motor deficits, similar to the phenotype of *PARK15* patients (73). Vingill et al. also showed that the loss of *FBXO7* affected the assembly of proteosomes leading to reduced proteosome activity. This is in keeping with dysfunction of the ubiquitin proteosome system being central to neurodegeneration (74, 75). *VPS35* (*PARK17*) has recently come to the fore as a cause of late-onset familial PD (76). How this gene contributes to human PD is unclear however it has been suggested that *VPS35* mutations lead to mitochondrial dysfunction (77), and impaired lysosomal and autophagy function [for review see (76)], all of which contribute to PD pathogenesis. To date the potential effects that mutations in either *FBXO7* or *VPS35* might have on astrocyte function remain unknown; further experiments would be required to elucidate this.

## Microglia and Their Role in Parkinson's Disease

Microglia are the resident immune cells of the CNS. They originate in the yolk sac where they develop from early myeloid precursor cells. During embryonic development, primitive microglia migrate into the developing neural tube where they proliferate and populate the CNS (78). Due to the BBB, microglia lead a relatively sheltered existence compared to peripheral macrophages, although their functions remain the same. Their role is to continuously survey the microenvironment and respond to both physiological and pathological changes. In their capacity as the first line of defence in the CNS, they identify and remove unwanted material such as cellular debris.

The physiological roles of microglia include synaptic pruning and phagocytosis of apoptotic cells (79, 80). They may also aid synapse formation by releasing neurotrophic factors (81, 82). However, these cells do not escape the disruption that occurs during PD; they become activated and assume an inflammatory phenotype. Their expression of pattern-recognition receptors (PRR) allows them to respond to the presence of pathogen-associated molecular patterns (PAMPs) and damage-associated molecular patterns (DAMPs) in the microenvironment thereby resulting in microglial activation. Sustained pro-inflammatory activation of microglia, i.e., microgliosis, has been implicated in the pathogenesis of many neurodegenerative disorders including PD. These activated microglia assume an amoeboid morphology, coupled with increased phagocytic capacity. They are also highly reactive and are associated with expression of inflammatory molecules, such as pro-inflammatory mediators and reactive species, in addition to receptors for antigen recognition such as TLR2 (2). Their physiological cellular processes are also thought to be disrupted as a result, further contributing to disease pathogenesis.

While the clinical characteristics of PD have been well-defined, the aetiology of idiopathic PD is still unknown and under investigation. Among the potential causes of PD which are being considered are inflammation and microglia, whose role in PD was first posited in 1988 (15). There is evidence of microgliosis in the SN of PD patients as a result of post-mortem studies where significant CD68 and Iba1 immunoreactivity was detected. Since Iba1 immunoreactivity was co-localised with TLR2 it is thought that this receptor may play a key role in microgliosis (83). This localised microgliosis is also present in the MPTP mouse model of PD (84), the A53T α-syn transgenic mouse model of PD following administration of LPS (85) and the L-dopa-induced dyskinesia rat model of PD (86). Blocking microglial activation with a combination of matrix metalloproteinase inhibitor 1-DNJ plus ibuprofen is protective against dopamine neuronal loss (87) suggesting that microglia are key contributors to PD pathology.

### The Impact of Parkinson's Disease Genes on Microglial Function

PD-associated genes are expressed by microglia, not just neurons and astrocytes. The products of these gene mutations are thought to affect the functioning of these cells (14), and can exacerbate microgliosis (Table 2). The dominant PD-risk genes *SNCA* and *LRRK2* promote neuroinflammation via activation of microglia and inflammatory signalling pathways such as NF-κB. Besides cell activation, PD-associated genes also disrupt other microglial processes including mitochondrial respiration and autophagy (99). Since autophagy is involved in regulating microglia inflammatory status (100, 101), its disruption has been reported to play a critical role in inflammation (102) and may also affect some of the key functions of microglia including phagocytosis (101). Furthermore, autophagy failure has been shown to promote intercellular propagation of α-syn (103) which in turn drives microglial activation and neuroinflammation. Mitochondrial dysfunction is another pathological feature in PD, as such the mitochondrial toxin MPTP, is commonly used to induce PD pathology in rodents in order to model disease pathology. Furthermore, pesticides paraquat and rotenone can also be used to induce parkinsonism via disruption of the respiratory chain in mitochondria (104). A number of genes associated with increased PD risk are linked to mitochondrial homeostasis. Among these are *SNCA, PARK2, PINK1, PARK7*, and *LRRK2* (104) (Table 2).

**Table 2:** The role of microglia and Parkinson's disease risk genes in Parkinson's disease pathology.

**Gene/Protein**	**Model used**	**Main findings**	**References**
*Park7*/DJ-1	DJ-1 knockdown microglia	• DJ-1 deficient microglia have impaired uptake of α-syn • DJ-1 deficient microglia exhibit impaired autophagy which in turn affects α-syn clearance • DJ-1 deficient microglia show increased inflammatory response to α-syn	(88)
*Park7*/DJ-1	DJ-1 knockout mice	• DJ-1 KO enhanced expression of ICAM-1, IFN-γ, IL-1β, IL-17 and I-TAC, and enhanced dopamine neuron loss in response to LPS • DJ-1 deficiency sensitises microglia to release IFN-γ and I-TAC via enhanced NF-κB signalling	(89)
*Park7*/DJ-1	N9 murine microglia cells + DJ-1 shRNA	• DJ-1 deficiency increases mitochondrial and MAO activity, and reduces migration in microglia • DJ-1 deficient microglia have increased ROS, NO, IL-6, and IL-1β production and secretion following LPS insult • DJ-1 microglia have increased neurotoxicity	(90)
*PINK1*/PINK1	Primary glia cultures	• PINK1-deficiency in microglia causes increased NO production and inflammatory gene expression following LPS/IFN-γ stimulation • PINK1-deficiency reduces IL-10 expression in primary microglia	(44)
*PARK2*/Parkin	BV2, N9 and primary microglia	• Parkin knockdown exacerbates pro-inflammatory response to LPS via over-activation of JNK and NF-κB pathways • Parkin silencing attenuates progression of necroptosis	(91)
*PARK2*/Parkin	*Park2* KO microglia	• PARK2-deficiency has a priming effect on microglia leading to enhanced activation and NLRP3 induction following LPS exposure; due to loss of A20 negative feedback regulation	(92)
*SNCA/*α-syn	α-syn PFF mouse model	• MHCII immunoreactive microglia co-localise with α-syn in SN • SN microglia exhibit reactive morphology	(93)
*SNCA/*α-syn	Primary mouse microglia + A53T mutant	• Intensity of microglia activation is dependent on the type of α-syn • A53T promotes ROS production in microglia • A53T induces STAT1 phosphorylation and activation of MAP kinases activation, and induces microglia reactivity via NF-κB, AP-1, and Nrf2 pathways	(94)
*SNCA/*α-syn	BV2 cells and primary microglia	• A53T mutant α-syn promotes PHOX activation in BV2 and primary microglia • α-syn is recognised by the P2X7 receptor which is necessary for α-syninduced PHOX activation via the PI3K/AKT pathway	(95)
*LRRK2*/LRRK2	LRRK2 KO mice + LPS + paraquat	• LRRK2 KO prevents microglia activation and TH^+^ neuron loss following intranigral injection of LPS and paraquat. Motor function is preserved. Mechanistic involvement of WAVE2 is proposed	(96)
*LRRK2*/LRRK2	Human iPSC-derived microglia	• IFN-γ increased LRRK2 expression in microglia • LRRK2 regulates function in microglia; *LRRK2-G2019S* mutant microglia had greater phagocytic capacity and decreased cytokine secretion • LRRK2 KO have defective glycolytic shift following stimulation with LPS	(97)
*LRRK2*/LRRK2	Human embryonic microglia	• Manganese increases LRRK2 expression and kinase activity • LRRK2 inhibition attenuates manganese-induced apoptosis, oxidative stress, TNF-α production, and MAPK signalling	(98)

*KO, knockout; I-TAC, interferon-inducible T-cell alpha chemoattractant; MAO, monoamine oxidase; ROS, reactive oxygen species; NO, nitric oxide; LPS, lipopolysaccharide; JNK, c-Jun N-terminal kinase; NLRP3, NOD-, LRR- and pyrin domain-containing protein 3; PFF, pre-formed fibrils; PHOX, nicotinamide adenine dinucleotide phosphate oxidase; P2X7, purinergic receptor; PI3K/AKT, phosphoinositide 3-kinase/protein kinase B*.

#### DJ-1/PARK7

DJ-1 is a sensor for oxidative stress which localises to the mitochondria when the cell is exposed to oxidative stress (90). DJ-1 mutations which lead to protein deficiency have been linked to PD, with 1% of hereditary cases of PD linked to this gene (88). DJ-1 deficient microglia demonstrate impaired uptake and degradation of α-syn, as well as impaired autophagy (88), and increased sensitivity to pro-inflammatory signals such as LPS (105, 106). This impairment of microglia function combined with increased sensitivity to inflammatory signals may leave PD patients with mutant DJ-1 prone to neuroinflammation. Monoamine oxidase is an enzyme which breaks down amine neurotransmitters such as dopamine. Monoamine oxidase inhibitors have been applied in combination therapy for PD and interestingly one of these, Rasagiline, has been shown to reduce the pro-inflammatory phenotype in microglia and subsequently reduce neurotoxicity in a DJ-1 knockout model (90). This suggests that Rasagiline may be particularly successful in the treatment of PD patients with *PARK7* mutations.

#### SNCA

Encoded by the *SNCA* gene, α-syn is the main component of Lewy bodies in PD. Physiological roles of α-syn include synaptic vesicle trafficking and formation of the SNARE complex for exocytosis of synaptic vesicle contents into the synapse (14, 107). In PD, excess and/or mutant α-syn aggregates form fibrils. These neurotoxic fibrils act as an endogenous DAMP causing activation of microglia via TLR2 (2, 94) resulting in the activation of NF-κB and MAPK pathways and the production and release of pro-inflammatory mediators such as TNF-α, IL-6, CCL5, and IL-1β (2, 36). Interestingly, microglia activation in response to α-syn has been demonstrated to be mutant specific (94) (Table 2). While α-syn and Lewy bodies are characteristic of PD, it is possible that patients with *SNCA* mutations are vulnerable to enhanced levels of microgliosis and neuroinflammation. α-syn is now thought to aggregate in microglia as well as in neurons. α-syn binding to surface FcγRIIB receptors on microglia inhibits phagocytosis (108), furthermore α-syn accumulation within microglia can disrupt phagocytosis and lead to activation of microglia (109). TLR2 has been identified as a receptor for α-syn, capable of transporting secreted α-syn into the cytoplasm of microglia (110). Numerous studies have demonstrated a role for α-syn in the activation of microglia, via TLR2 (111–113) and TLR4 (114). Besides TLR2 and TLR4, uptake of α-syn by microglia is facilitated by FYN kinase and CD36 (115). Internalised α-syn can act as a priming signal for the NLRP3 inflammasome, and also disrupts mitochondria function leading to the production of ROS which are capable of activating the NLRP3 inflammasome (115).

#### LRRK2

*LRRK2* encodes the leucine-rich repeat serine/threonine-protein kinase 2. *LRRK2* is an incompletely penetrant gene associated with increased PD risk (116, 117). The *LRRK2-G2019S* mutation has a variable penetrance and is associated with both sporadic and familial PD. There is evidence that LRRK2 is capable of regulating macrophage and microglial motility and phagocytosis (118), mediated by RAB10 (119, 120). WAVE2, a novel interacting partner with LRRK2, regulates branched actin and the Rac1 effector molecule thereby promoting actin polymerisation and cytoskeletal reorganisation. This rearrangement of the cytoskeleton is crucial for the phagocytotic functions of myeloid cells (121). WAVE2 is proposed to be regulated by LRRK2, therefore LRRK2 deficiency can compromise this protein and subsequently reduce the phagocytic capacity of microglia (96, 121) (Table 2). Mutated LRRK2 is observed to alter mitochondrial morphology and increase mitochondrial fission in microglia. This was demonstrated via increased levels of Drp1, a mitochondrial fission marker, CD68, a microglia activation marker and TNF-α in LRRK2 mutant mice (122). Furthermore, inhibition of LRRK2 has been shown to attenuate microglial inflammatory response to TLR4 stimulation (123), and activation of microglia by extracellular α-syn (124, 125).

#### Other Parkinson's Disease Risk Genes

Besides the main culprits, there are many other genes associated with PD which are proposed to affect microglia function. *GBA* encodes the lysosomal hydrolase GCase; an incompletely penetrant gene associated with increased PD risk. There is limited literature pertaining to GBA microglia mutants in the PD context, the majority of papers discuss Gaucher disease. For example, in zebrafish, GBA knockout leads to early microglial activation, reduced motor activity, loss of dopaminergic neurons, and ubiquitin inclusions (126). Furthermore, GBA mutations can result in accumulation of glucosylceramide and complement activation which in turn drive inflammation (127).

Recent studies have revealed that autophagy dysfunction can be closely linked with PD pathogenesis. ATG5 (autophagy-related protein 5) is an essential component of the autophagosome, and deficiency results in impaired autophagy. A number of ATG5 variants have been found in the PD patient population (99, 128).

*PINK1* and *PARK2* have been linked to autosomal recessive forms of PD (116). PINK1 deficiency in microglia has been linked to increased production and secretion of pro-inflammatory mediators, coupled with a reduction in the production and secretion of anti-inflammatory factors thereby favouring a reactive phenotype in microglia (44). The same phenotype is also associated with *PARK2* (Table 2). Deficiency in the associated protein Parkin, achieved via knockdown in microglial cells, has a priming effect and results in an enhanced response to the pro-inflammatory stimulus LPS (91, 92). PD patients with these genes may have greater levels of microgliosis as a result.

There is a clear role of microglia in the pathogenesis of PD and the major PD risk genes are a driving force behind this. However, it is becoming clear that microglia are not the only immune cells involved in PD. It is very well-established that the BBB is compromised in PD therefore allowing immune cells from the periphery to infiltrate the CNS.

## An Emerging Role for the Peripheral Immune System in Parkinson's Disease

Evidence of an important role for inflammation in the pathogenesis of PD is emerging and the data suggests that peripheral immune cells may contribute to this inflammation. In this section of the review we cover the latest studies demonstrating a role for the peripheral immune system and in particular T cells in PD. By way of introduction however a brief explanation of innate and adaptive immunity is outlined below.

The immune system is divided into the innate and adaptive arms which cooperate to defend against infection, however when dysregulated, immune responses can be important contributors to diseases. The innate immune system represents the body's first line of defence against invading pathogens, it is comprised of numerous cell types which carry out functions such as phagocytosis and antigen presentation. Innate immune cells include dendritic cells, macrophages and also microglia, which can be activated via PRR recognising not only PAMPS but also endogenous DAMPS including α-syn (129). On the other hand, adaptive immunity consisting of B and T lymphocytes provides a highly specific, targeted response capable of dealing with a variety of different intracellular or extracellular infections. T cells, which are either CD4^+^ or CD8^+^ are initially naïve until their T cell receptor (TCR) recognises its specific antigen presented by antigen presenting cells via MHC molecules (130). In general, endogenous antigens such as those from viruses are presented via MHCI to CD8^+^ T cells whereas exogenous antigens are presented via MHCII to CD4^+^ T cells (130). All cells in the body express MHCI and can activate CD8^+^ T cells whereas only professional antigen presenting cells including dendritic cells, macrophages, B cells and microglial cells express MHCII and have the capacity to activate CD4 T cells. Once initially activated in the secondary lymphoid organs, naïve T cells differentiate into effector cells with specific functionalities tailored to the infection. CD4^+^ T cells differentiate into one of the T-helper subtypes; Th1, Th2, Th17 cells which produce various cytokines and provide help to B cells (130). CD8^+^ T cells differentiate into cytotoxic T lymphocytes which induce apoptosis of infected cells without affecting adjacent healthy cells. Naïve B cells, once activated by their specific antigen and with help from T helper cells, differentiate into plasma cells, producing antibodies which specifically target the antigen and promote clearance via phagocytosis (130). Once activated and differentiated in the lymphoid organs T and B cells traffic to the tissues where they become reactivated upon encounter of an antigen and carry out their effector functions (130). Thus, the immune system has evolved to deal effectively with infection, however various genetic and other factors can conspire to result in inappropriate or chronic inflammation in response to altered proteins or self-antigens that manifests in autoimmune and inflammatory disease (131). Neuroinflammation associated with disorders such as Alzheimer's disease and PD attracts peripheral immune cells to the CNS, and the disruption of the BBB which is a pathological feature of these diseases means that there is a pathway for these cells to enter the brain parenchyma (132–134).

### A Potential Role for T Cells in Parkinson's Disease

In the last decade there has been mounting evidence of a role for T cells in the pathogenesis of PD (Table 3). Despite their important role as part of the adaptive immune system there is little evidence of the involvement of B cells in PD. Brochard et al., identified both CD8^+^ and CD4^+^ T cells but not B cells or natural killer cells in the post-mortem brain tissue of PD patients (138). The presence of both CD8^+^ and CD4^+^ T cells was also evident in the MPTP and α-syn overexpressing mouse models of PD (138, 147). However, Theodore et al., observed both infiltrating B and T cells following injection with α-syn overexpressing adeno-associated viral vector (AAV) into the SN of mice (151). The differences with respect to the possible role of B cells in these studies may be attributed to the study model and human PD tissue vs. animal model, however more research is required before a definitive conclusion can be drawn.

**Table 3:** Evidence for the role of T lymphocytes in Parkinson's disease.

**Specimens**	**Study groups**	**Main findings**	**References**
Human PBMCs and plasma	PD patient and HS	• Decreased levels of Tregs observed in PD patients compared to controls • No significant difference in Th1, Th2, and Th17 levels between patients and control, however serum levels of IL-17A were decreased in PD patients • Serum levels of pro-inflammatory cytokines TNF-α, IL-1β, IL-6, and GM-CSF not significantly different between groups	(135)
Human whole blood	PD patient and HS	• Overall lymphocyte numbers were reduced • CD4^+^ T cell levels were reduced, CD8^+^ T cells increased • Treg cells were significantly reduced • IL-4 producing cells were significantly reduced, IFN-γ/IL-4 ratio was significantly increased	(136)
Human whole blood Rodent *in vivo*	PD patient and HS MPTP^+^, MPTP^−^ 6-OHDA^+^, 6^−^OHDA-	• Decreased lymphocyte numbers, both B and T cells were reduced • CD4^+^ T cells decreased, CD8^+^ T cells were consistent • Significant increase in activated CD4^+^ T cells and reduction in naïve and memory CD4^+^ T cells • MPTP, but not 6-OHDA treatment, induced activation of CD4^+^ T cells	(137)
Human post-mortem tissue Rodent *in vivo*	PD patient and HS MPTP^+^, MPTP^−^ Tcrb^−/−^, Rag1^−/−^, CD4^−/−^, CD8^−/−^	• Post-mortem tissue of PD patients and MPTP-mouse model demonstrates infiltration of CD8^+^ and CD4^+^ T cells in PD • MHCI expression was observed on dopamine neurons of the SN in PD post-mortem samples • MPTP-induced neurodegeneration decreased in the absence of T cells and cell death was attenuated by a lack of CD4^+^ T cells not CD8^+^ T cell	(138)
Human post-mortem tissue and hESC	PD patient and HS	• Microglia conditioned media from α-syn and neuromelanin activated microglia cause expression of MHCI in Vm-neurons • Vm-neurons are capable of inducing proliferation of cytotoxic T lymphocytes which in turn cause neuronal cell death	(139)
Rodent *in vivo, in vitro* primary microglia and CD4^+^ T cells	AAV2-Syn or AAV2-GFP and WT or MHCII^−/−^	• Overexpression of α-syn causes increased expression of MHCII on microglia • Knockout of MHCII attenuates α-syn-induced microglial activation in the SN pars compacta and dopaminergic cell loss	(140)
Human whole blood and isolated PBMCs	PD patient and HS	• PD patients demonstrate lower absolute counts but not frequency of Th17 cells and Tregs • PHA stimulation caused greater increase in IFN-γ and TNF-α in PD patients than HS, however no difference in IL-17A was observed and IL-10 was increased in HS but not in PD patients relative to non-stimulated cells • Co-culture of Teff and Treg cells caused ~80% reduction of IFN-γ and TNF-α in HS but only ~20% in PD patients	(141)
Human PBMCs	PD patient, AD patient and HS	• Increased α-syn specific T-cell reactivity prior to PD diagnosis declining post-diagnosis • Increased T cell reactivity in response to α-syn in PD patients compared to AD patients and HS	(142)
Rodent, *in vivo, in vitro* neurons and T cells	MPTP^+^ and MPTP^−^	• MPTP^+^ mice demonstrate BBB disruption and infiltration of Th17 cells in SN • IL-17, IL-1β, TNF-α, iNOS, IL-22, and IFN-γ increase in the SN of MPTP^+^ mice, BDNF and GDNF decrease • Co-culture of Th17 cells with Vm-neurons causes increased TNF-α and IL-1β, and induces neuronal cell death via LFA-1/ICAM-1	(143)
Rodent, *in vivo, in vitro* microglia, neurons and Th17 cells	MPTP^+^ and MPTP^−^	• MPTP caused BBB disfunction and increased IL-17A in SN only • Teff cells increase the frequency of CD4^+^ T cells, reduce TH^+^ cell numbers in the SN, decrease dopamine levels in the striatum and increase IL-1β and TNF-α levels in MPTP mice • Knockout of IL-17A alleviates these effects • IL-17A-induced neuronal cell death does not occur in the absence of microglia • Silencing IL-17A receptor on microglia prevents IL-17-induced cell death	(144)
Rodent, *in vivo, in vitro* Treg, Teff and microglia	MPTP^+^ and MPTP^−^	• Adoptive transfer of Treg attenuates MPTP-induced microglial activation and neuronal cell loss • Adoptive transfer of Treg increase neurotrophic factors; BDNF and GDNF	(145)
Rodent, *in vivo, in vitro* CD4^+^ T cells	MPTP^+^ and MPTP^−^	• α-syn induced Th1/Th17 cell phenotypes from naïve T cells • Adoptive transfer of α-syn stimulated Th1 and Th17 cells caused neuronal death in the SN, and increases cell death observed in MPTP model	(146)
Rodent, *in vivo*	WTS^+^/Rag2^+^, WTS^+^ /Rag2^−^, WTS^+^/Rag^−^ and WTS^−^/Rag2^+^	• Increased levels of insoluble α-syn in Rag^+^ mice compared to Rag^−^ mice • CD4^+^ and CD8^+^ T cells observed in the brain of WTS^+^/Rag^+^ mice but not in WTS^+^/Rag^−^ or WTS^−^	
		• M1 phenotype prominent in WTS^+^/Rag^−^ mice compared with an M2 phenotype in WTS^+^/Rag^−^ mice which demonstrate increased phagocytosis of α-syn	(147)
Human iPSC and T cells	PD patient and HS	• Increased frequencies of IL-17 producing CD4^+^ T cells in PD patients, no significant difference in IFN-γ or IL-4 producing cells • Co-culture of iPSC-midbrain neurons with Th17 cells/IL-17 increased neuronal cell death and levels of IL-17, IL-1β, TNF-α, and IL-6 in PD cells • Neuronal cell death in PD co-cultures occurred via IL-17/IL-17 receptor signalling, and potential activation of the NF-κB signalling pathway and preventing IL-17/IL-17 receptor interaction attenuated this	(148)
Human whole blood	PD patient and HS	• Reduced levels of B and T lymphocytes in PD patients	(149)
Human isolated PBMCs	PD patient and HS	• α-syn peptides presented by both MHCI and MHCII induce T cell proliferation in PD patients • T cells mainly either IFN-γ or IL-5 producing	(150)
Rodent, *in vivo*	AAV2-Syn or AAV2-GFP	• α-syn overexpression caused increased expression of CD68, IgG deposition and increased ICAM-1, IL-6, IL-1α, and TNF-α levels • COX-2 remained unchanged and iNOS expression decreased at a later time point • Increased levels of CD3^+^ and CD45R^+^ cells following α-syn overexpression	(151)

*6-OHDA, 6-hydroxydopamine; Teff, T effector cells; Vm, ventral midbrain; HS, healthy subjects; PHA, phytohemagglutinin; MPTP, 1-methyl-4-phenyl-1,2,3,6-tetrahydropyridine; Rag, recombination activation gene; Tcrb, T cell receptor beta; PBMC, peripheral blood mononuclear cell; iNOS, inducible nitric oxide synthase; COX-2, cyclooxygenase 2; AAV2, adeno-associated viral vector, serotype 2; WTS, human wild type α-syn overexpressing*.

Disruption of the BBB is commonly observed in PD pathogenesis, presenting an opportunity for peripheral immune cells to infiltrate into the brain. Infiltration of T cells from the periphery into the CNS has been observed in multiple PD studies (132, 144). Liu et al., demonstrated using MPTP treatment that BBB disruption increases the frequency of CD4^+^ T cells in the SN of mice (144). The infiltration of CD4^+^ T cells reduced the number of TH^+^ neurons, decreased dopamine levels and increased the production of IL-1β and TNF-α. Importantly, Brochard et al., observing both CD8^+^ and CD4^+^ T cell infiltrates, determined using CD4^−/−^ and CD8^−/−^ mice that CD4^+^ T cells, rather than CD8^+^ T cells, were responsible for the neurodegeneration associated with their infiltration (138). As discussed above, the accumulation of misfolded α-syn protein contributes to dysregulation and inflammation in PD (152). Studies have demonstrated that T cell infiltration is associated with α-syn overexpression (147, 151), additionally the knockout of lymphocytes reduced α-syn protein aggregates in this PD model (147). These studies indicate a role for T cells in PD, however given that T cells are activated in an antigen-specific manner via MHC molecules, it is essential to understand the antigen specificity of T cells involved in PD and the possible contribution of different MHC haplotypes.

#### The Role of MHC and Antigen Presentation in Parkinson's Disease

**Figure 1:**
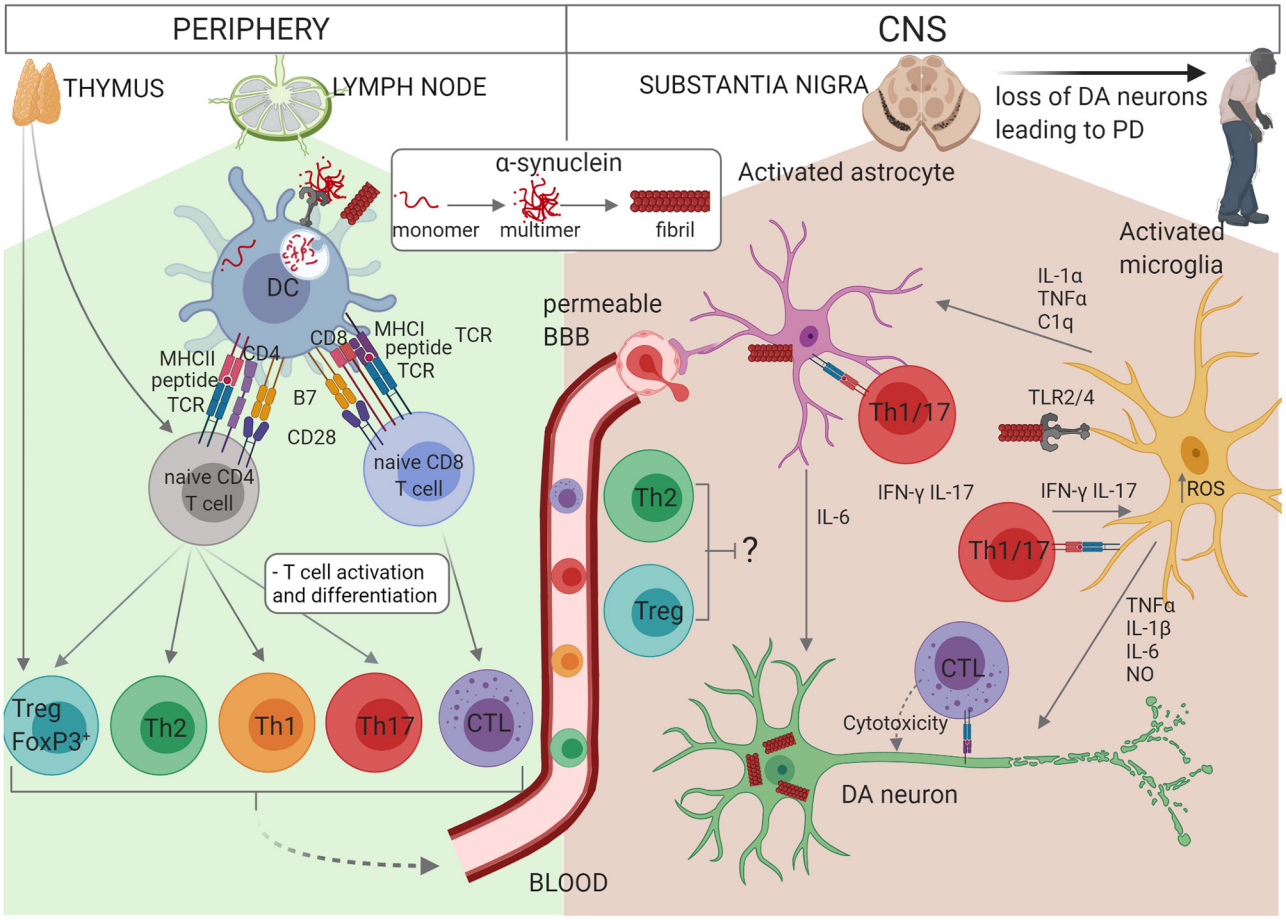
Schematic representation of the interactions between glial cells and immune cells in PD. Self-reactive α-syn-specific naïve T cells may have escaped thymic selection, or alternatively post-translationally modified α-syn may be recognised by naïve T cells as a neoantigen. In individuals with *PINK1/PARK2* mutations, mitochondrial antigen presentation may also result in T cell activation. Autoreactive CD4^+^ or CD8^+^ T cells circulate through the lymph nodes where they may become activated by a dendritic cell (DC) presenting α-syn antigen via MHCII or MHCI, respectively. α-syn or other DAMPs activate the DC via PRR to express co-stimulatory molecules (B7) and cytokines which drive the proliferation and differentiation of effector T cell subsets. CD4^+^ T cells differentiate into Th1, Th2, Th17, or Treg cells depending on the cytokine milieu and CD8^+^ T cell differentiate into cytotoxic lymphocytes (CTL). Effector T cells traffic via the blood and extravasate through a permeable BBB into the CNS, where they re-encounter their α-syn antigens presented via MHCI on neurons or MHCII on astrocytes or microglia. Th1 and Th17 cells produce pro-inflammatory cytokines IFN-γ and IL-17 which contribute to the activation of astrocytes and microglia in synergy with other cytokines such as TNF-α, and CTL induce apoptosis in DA neurons. On the other hand, Treg and Th2 cells may protect against neuroinflammation. The accumulation of modified or aggregated α-syn is thought to be a key initiator of PD. α-syn accumulates within DA neurons and can also be secreted where it activates astrocytes and microglia. Activation of TLR2 or TLR4 by α-syn together with increased intracellular ROS activates the inflammasome. Activated microglia secrete TNF-α, IL-1β, IL-6, and NO which promote DA neuron degeneration. IL-1α, TNF-α, and C1q from activated microglia also activate astrocytes which in turn secrete IL-6, IL-1α, IL-1β, and NO. In addition, activated astrocytes exhibit decreased release of protective neurotrophic factors and impaired glutamate uptake. Thus, T cells, astrocytes and microglia cooperate to perpetuate neuroinflammation and loss of DA neurons in PD.

In addition to the evidence above showing the presence of T cells in the SN of PD patients and mouse models, a role for T cells is also implied by the identification of specific MHC haplotypes and non-coding SNPs in MHC genes as risk factors for PD. Such MHC associations are a common feature of autoimmune diseases driven by auto-reactive T or B cells and are thought to result from preferential presentation of dominant self-epitopes by particular MHC molecules to auto-reactive T cells that have escaped thymic selection. Interestingly, Sulzer et al., demonstrated significantly enhanced T cell responses to immunodominant α-syn peptides in PD patients, relative to healthy controls (150). Furthermore, an immunodominant peptide was shown to bind with high affinity to the DRB1^*^15:01 and DRB5^*^01:01 alleles which were part of the MHC haplotype previously associated with PD (150). In a subsequent study, Lindestam Arlehamn et al., determined in a single longitudinal case study that this α-syn-specific T cell response was elevated prior to diagnosis and decreased thereafter (142). In the studies above, not all PD patients exhibited α-syn-specific T cell responses and responses were also observed in some healthy controls, indicating that in both PD and healthy controls these auto-reactive T cells escaped deletion during thymic selection. However, such auto-reactive T cells only become pathogenic if peripheral tolerance mechanisms are overcome and they become activated in the secondary lymphoid organs upon recognition of α-syn peptides presented via MHC molecules on activated dendritic cells (Figure 1). Tolerance can be overcome when self-antigens become post-translationally modified and then appear as neoantigens to which the immune system is not yet tolerised. Indeed, T cell responses to both native and post-translationally modified α-syn were observed in PD patients (150). The α-syn that activates naïve T cells in PD could either be peripherally derived or have drained from the CNS as suggested by the proposed body-first vs. brain-first subtypes, respectively (153). In addition, activation of the antigen presenting cell via PRR is required in order to induce the necessary co-stimulation signals required for naïve T cell activation and the source of this signal is unclear but could possibly be provided via DAMPs including α-syn.

#### Mitochondrial Antigen Presentation in Parkinson's Disease

Recent studies in a mouse model have identified an intriguing link between the PD-associated genes *PINK1* and *PARK2* and antigen presentation. *PARK2* and *PINK1*, which cause autosomal recessive forms of PD (116), maintain mitochondrial homeostasis through mitophagy (110). Loss of function mutations in the *PINK1* gene (a mitochondrial kinase) lead to disturbances in mitophagy, and mitochondrial fusion and fission (154). PINK1 deficiency is associated with increased ROS production, oxidative stress, abnormal mitochondrial function, and altered morphology (44). Mitochondrial dysfunction can result in the release of numerous pro-inflammatory factors such as mitochondrial DNA, RNA, ATP, and cytochrome c (110). The presence of ROS and DNA from damaged mitochondria can act as danger signals for activation of the NLRP3 inflammasome which is involved in neuroinflammation in PD (2, 155, 156). Mitochondrial dysfunction has been shown to over-activate the NLRP3 inflammasome in microglia resulting in dopaminergic neuron cell death (157). In its capacity as an activator of ubiquitin PINK1 cooperates with Parkin a ubiquitin ligase (158, 159). In addition to these established roles in maintaining mitochondrial homeostasis, PINK1 and Parkin have more recently been shown to play a role in suppressing immune responses by actively inhibiting the presentation of self-mitochondrial antigens via MHCI (160). Mitochondrial derived vesicles can arise by budding from the mitochondria and then fuse with lysosomes, processes that are dependent on the proteins SXN9 and RAB7 GTPase. Mitochondrial antigens are then processed and presented via the MHCI pathway to activate autoreactive CD8^+^ T cells. PINK1 and Parkin however inhibited the activity of SXN9/RAB7 and thereby mitochondrial antigen presentation (160). These data suggest that familial PD associated with loss of function of PINK1/Parkin could involve an autoimmune component, whereby the presentation of self-mitochondrial antigens is enhanced in the absence of PINK1 or Parkin resulting in the activation of autoreactive CD8^+^ T cells. These mechanisms were demonstrated in dendritic cells, macrophages, and fibroblasts; however, a key outstanding question is whether lack of PINK1/Parkin exerts similar effects on mitochondrial antigen presentation in astrocytes, microglia and neurons. If so, then naïve CD8^+^ T cells could be initially activated in the periphery by dendritic cells, traffic to the CNS and cross the BBB where they would kill dopaminergic neurons upon recognition of their cognate mitochondrial antigen presented via MHCI. Furthermore, it is also unknown whether the absence of PINK1/Parkin could result in mitochondrial antigen presentation via MHCII to activate CD4 T cells. This is an important question given that neurodegeneration was shown to be dependent on CD4 T cells in a mouse model of PD (138). Interestingly mitochondrial antigen presentation was shown to be enhanced by an inflammatory insult and indeed in a follow up study, Matheoud et al. demonstrated that intestinal infection in PINK1 deficient mice promoted mitochondrial antigen presentation, the activation of CD8^+^ T cells specific for mitochondrial antigens and motor impairment that was reversed by L-DOPA (161). Given that loss of function of PINK1 or Parkin results in symptoms of PD in humans but not mice (162), this suggests that it is a combination of the genetic mutation together with an inflammatory insult that leads to PD in individuals with mutations in *PINK1/PARK2*. Importantly, a role for the gut-brain axis in PD is implicated by these findings and supported by a study that showed that the gut microbiome was required to induce disease in an α-syn mouse model of PD. Furthermore, transfer of microbiota from PD patients into α-syn overexpressing mice resulted in exacerbation of disease symptoms when compared with transfer of microbiota from healthy controls (163). These studies suggest that dysbiosis could be responsible for triggering or exacerbating disease at least in some familial cases of PD and raise the interesting possibility of preventative or therapeutic targeting of the microbiome.

In summary, the evidence from post-mortem and MHC association studies in PD patients together with that from murine studies, suggests involvement of T cells in the pathogenesis of PD. However, there are still many unanswered questions and more extensive research is required. For example, to translate the autoimmune hypothesis of PD described above it will be important to identify mitochondrial antigen-specific T cells in PD patients with *PINK1/PARK2* mutations. The possible role of different T cell subtypes in PD is discussed below.

#### Th1 Cells in Parkinson's Disease

Th1 cells differentiate under the influence of cytokines IFN-γ and IL-12 released by antigen presenting cells, in combination with activation of the T-bet transcription factor. The main cytokines released by Th1 cells are IFN-γ and TNF-α, these cells are important in activation of B cells and increasing phagocytosis of microbes (130). PD studies have observed both increased frequencies of IFN-γ-producing Th1 cells in circulating blood from PD patients (136, 141), and no significant difference in Th1 levels (135). Kustrimovic et al., discovered decreased levels of the T-bet encoding gene, *TBX21* (141, 164) in PD patients compared with controls. However, naive CD4^+^ T cells treated with α-syn polarised to a Th1 or Th17 phenotype causing cell death of TH^+^ neurons in the SN and exacerbated MPTP-induced cell death (146). The evidence above suggests pathogenic potential for Th1 cells in PD, however the evidence is also sparse and conflicting, which clearly highlights the need for further research in this area.

#### Th17 Cells in Parkinson's Disease

A large amount of research exists into the role of Th17 cells in PD. TGF-β, IL-6, IL-1, and IL-23 along with the RORγt transcription factor are important in inducing differentiation of Th17 cells (130, 165). The signature cytokine produced by Th17 cells is IL-17. Upon binding to its receptor, which is widely expressed on epithelial and other cell types, it induces the release of chemo-attractants such as CXCL1 to recruit additional immune cells, particularly neutrophils. IL-17 also induces the secretion of anti-microbial peptides such as S100A8/A9 (166). Sommer et al., observed increased frequencies of IL-17 producing CD4^+^ T cells in the peripheral blood of PD patients compared with control subjects (148) and infiltration of Th17 cells into the SN has been demonstrated in an MPTP mouse model of PD (143). In contrast however, a study using peripheral blood mononuclear cells (PBMCs) isolated from PD patients and healthy subjects discovered lower absolute counts but not frequency of Th17 cells in PD (141). A separate study observed no difference in the levels of Th17 cells between PD patients and controls, with reduced IL-17A levels in PD patients (135, 141).

Although the conflicting studies above indicate that further work is required to determine whether enrichment of peripheral Th17 cells is a consistent finding in PD, there is experimental evidence to suggest a pathogenic role for Th17 cells in the context of PD. Stimulation of Th17 cells with α-syn was found to cause neuronal cell death in the SN in an MPTP mouse model of PD (146). Furthermore, co-culture of MPTP-treated neurons with Th17 cells further exacerbated neuronal cell death and increased IL-1α and TNF-α levels observed with MPTP-treatment alone (143, 146). Liu et al., determined that these effects were mediated via lymphocyte function-associated antigen 1 (LFA-1) and intracellular adhesion molecule-1 (ICAM-1) interactions and ablation of either ICAM-1 or LFA-1 attenuated the dopaminergic cell death observed in this model (143).

Importantly, Sommer et al., demonstrated that the co-culture of autologous iPSC-midbrain neurons and Th17 cells led to increased neuronal cell death in cells derived from PD patients compared with controls and this effect was not observed with non-autologous cell cultures (148). This suggested that antigens were presented in an MHC-restricted manner by PD neurons to Th17 cells which then induced neuronal death. It was determined that either IL-17 or the IL-17 receptor signalling and a potential activation of NF-κB signalling was responsible for the neuronal cell death observed in the co-cultures. Furthermore, attenuation of neuronal cell death in the PD co-cultures was achieved by blocking the IL-17 or the IL-17 receptor. Taken together the above studies indicate a role for Th17 cells in the pathogenesis of PD. Although much of the research to date has been undertaken in animal models, future studies like that of Sommer et al., could utilise human-based models of PD, and patient-derived models to determine whether these interactions translate to the human disease phenotype.

#### Th2 Cells in Parkinson's Disease

Th2 cells differentiate from naïve T cells under the influence of IL-4, and the activation of the GATA3 transcription factor. These cells produce IL-4, IL-5, and IL-13 (130). IL-4 activates IgE production causing mast cell degranulation, releasing histamines, and other pro-inflammatory cytokines. Additionally, IL-4 as well as IL-13 increase mucous production and IL-5 activates eosinophils, releasing various pro-inflammatory and cytotoxic proteins (167). In the context of neuroinflammation unlike inflammatory Th1 and Th17 cells, Th2 cells are usually considered to be anti-inflammatory and protective. Alvarez-Luquin et al., demonstrated no significant difference in Th2 cell counts in PD patients compared with controls, however there was a significant increase in IL-13 observed (135). In contrast, studies have observed lower absolute numbers and frequency of Th2 cells compared with the healthy subjects (136, 141), however, increased GATA3 levels were identified (141). Sulzer et al., observed that α-syn peptides activated mainly IFN-γ-producing Th1 and IL-5-producing Th2 cells. As anti-inflammatory, protective immune cells Th2 cells have the potential to alter the progression of PD pathology. However, as observed with Th1 and Th17 cells the existing evidence is often contradictory, and therefore inconclusive, indicating the necessity for further research into this area before any concrete conclusions can be drawn.

#### T Cell Regulation in Parkinson's Disease

It is well-established that an overactive, aberrant immune response is rooted in the pathogenesis of numerous inflammatory diseases. Therefore, proper functioning of the regulatory mechanisms is key to preventing pathology. CTLA-4 is a crucial regulator of T cell activation, upon activation T cells upregulate the expression of CTLA-4, its role is to preferentially bind the co-stimulatory B7 in place of CD28, having an inhibitory, rather than stimulatory effect (130, 168, 169). Importantly, Cook et al., discovered a reduction in CTLA-4 expression on the surface of T cells from PD patients following stimulation (169), indicating the possibility of a potentially unregulated T cell response occurring in PD.

Regulatory (Treg) cells play a key role in constraining effector T cells and prevent excessive inflammation and autoimmunity. Treg cells are either directly generated early in life in the thymus during T cell development or differentiate in the periphery from naïve CD4^+^ T cells under the influence of TGF-β (130, 165) and they express the transcription factor FOXP3 which is necessary for their function. These cells produce IL-10 and TGF-β, which are important anti-inflammatory cytokines crucial to the regulation of the immune response and can also suppress effector T cells via various other mechanisms including via CTLA-4 (130). Reduced absolute numbers but not frequency of Treg cells have been observed in PD patients (135, 136, 141). Kustrimovic et al., also observed increased mRNA levels of FOXP3. Interestingly, T effector cells co-cultured with Treg cells only reduced IFN-γ and TNF-α by ~20% in PD patients, compared to an ~80% reduction observed in healthy controls, suggesting that Treg suppression in PD may be impaired (141). Reynolds et al., demonstrated a neuroprotective role for Treg cells in the MPTP mouse model of PD. The Treg cells were adoptively transferred into MPTP treated mice, a reduction in neuronal cell death, microglial activation, and increased production of both BDNF and GDNF were observed (145). Thus, Treg cells are likely to be protective in the context of PD and although there are some discrepancies, these data suggest that Treg cells may be numerically and or functionally impaired in PD and this may contribute to neuroinflammation.

The literature supporting a role for T cells in PD is growing, although there still remains evidence which contradicts this, as numerous studies have observed reduced levels of circulating T cells in the blood of PD patients compared with healthy subjects (136, 137, 141, 149). Similarly, most studies on T cell subsets presented contrasting findings. The discrepancies between studies may be explained in part by different methodologies; some studies have identified Th subsets via their production of signature cytokines whereas others used a combination of chemokine receptors. Similarly, the markers used to identify Treg cells were not always comparable between studies and identification of Treg cells is notoriously difficult as some of the markers used can also be induced on non-Treg cells during inflammation. For this reason, a full panel of markers including CD4, CD25, CD127, and FOXP3 is required to most reliably identify Treg cells. Finally, it is worth noting that alterations in the frequency of peripheral T cell subsets may not reflect changes occurring in the inflamed tissues and a reduction observed in the periphery may be due to trafficking to the tissue. Thus, in summary, this area requires a greater amount of research before the precise role of these cells can be elucidated.

This review has outlined evidence demonstrating the role of microglia, astrocytes and even peripheral immune cells in PD pathogenesis. Logically, this leads to the question of whether there is potential interplay between these infiltrating peripheral immune cells and the resident microglia and astrocytes of the CNS in PD.

## The Interplay Between Glia and Peripheral Immune Cells in Parkinson's Disease

Glial cells are crucial to the maintenance of homeostasis within the CNS and the disruption of this is linked to numerous CNS diseases. There appears to be little evidence available outlining potential interactions between peripheral immune cells and glia in PD, however the research into this area is increasing and is summarised in Figure 1. A recent study demonstrated antigen presenting capabilities in astrocytes. MHCII expressing astrocytes were identified in close proximity to CD4^+^ T cells in the post-mortem brain tissue of PD patients and cultured human astrocytes exposed to pre-formed fibrils of α-syn expressed the T cell co-stimulatory structures, CD80, CD86, and CD40 (170), suggesting the capacity to activate CD4^+^ T cells. Although Rostami et al., determined that cultured human microglia demonstrated poor antigen presenting capabilities, Harms et al., determined in mice that microglia exposed to α-syn increased their expression of MHCII, became activated and induced proliferation of CD4^+^ T cells. Furthermore, the knockout of MHCII prevented microglial activation and dopaminergic cell loss in these mice (140).

ICAM-1 is present on immune cells and its binding to LFA-1 is key to the migration, extravasation and even activation of immune cells (171). Prajeeth et al., discovered that it is possible for Th1 and Th17 cells to induce reactive, pro-inflammatory astrocytes which microglia migrate toward and increase their phagocytic ability. In addition to this, the T cell activated astrocytes enhanced the infiltration of Th17 cells (172). In other neurodegenerative diseases, such as Alzheimer's disease, astrocytes expressing ICAM-1 have been observed in close proximity to LFA-1 positive microglia (173). Miklossey et al., discovered the occurrence of these key immune interactions in post-mortem brain tissue from PD patients. ICAM-1 expression by astrocytes in the SN of PD patients was observed, and co-localised to these areas were LFA-1 positive microglia and LFA-1 positive leukocytes (174), highlighting the presence of interactions between glia and immune cells in PD.

The ability of these immune cells to infiltrate into the brain in PD is demonstrated in numerous studies. BBB disruption in PD has been observed in both post-mortem studies and animal models of PD (132, 144) and is linked to increased infiltration of peripheral immune cells (144). Once the immune cells enter the brain parenchyma their functions include the release of various cytokines, which are capable of activating glial cells and causing neuronal cell death (175). Liu et al., observed increased levels of IL-17A following BBB disruption in the MPTP mouse model of PD. Addition of IL-17A to co-cultures of microglia and neurons resulted in microglial activation, TH^+^ neuronal cell death and a decrease in dopamine levels and the inhibition of the IL-17A receptor on microglia was sufficient to attenuate these effects (144). The synergistic and additive effect of IL-17 with factors commonly secreted by astrocytes and microglia, such as IL-6, IL-1β, and TNF-α has been an area of great interest and numerous studies have demonstrated this phenomenon (176–180). The studies discussed in this review support the idea that inflammation is a key factor in the pathogenesis of PD and may be driven or compounded by T cells. However, there are important outstanding questions regarding the specificity of T cells involved in PD and whether auto-reactive T cells are involved only in certain subsets of PD patients such as those with genetic mutation, or whether they are more widely involved in sporadic PD. The role of the adaptive immune system in PD is a novel area of research and as such requires deeper investigation before a complete picture can be drawn of the role of these cells in PD pathogenesis and their therapeutic potential can be fully realised.

To aid in this, novel human-based models such as patient-derived iPSC and 3D culture systems should be utilised to determine if these findings from animal models translate to the human disease. As well as this, more patient studies will be required to determine what is occurring physiologically within PD patients and if this corresponds to what is observed within animal and *in vitro* human models.

## Implications for Therapeutic Targeting

Our increased understanding of the critical role the immune system plays in PD has stimulated research into the viable therapeutic targets it presents; so as to allow determination of their potential as disease modifying therapies. As mentioned earlier, Yun et al., demonstrated that targeting the GLP-1 receptor with NLY01 prevented conversion of astrocytes to a pro-inflammatory phenotype by activated microglia and inevitably reduced neuronal cell death (36). As a long-acting GLP-1 receptor agonist, with the ability to cross the BBB, NLY01 is currently undergoing phase 2 clinical trials to investigate its efficacy in early PD (NCT04232969). Although results have yet to be published for NLY01, another GLP-1 receptor agonist previously FDA approved for treatment of type 2 diabetes mellitus, Exenatide, is currently in phase 3 clinical trials (NCT04154072) having successfully completed early phase 1 and 2 trials (181).

A second newly emerging PD therapeutic target is that of monoclonal antibodies, specifically those targeting α-syn. PRX002/RG7935, an IgG 1 monoclonal antibody targeting aggregated α-syn, has demonstrated safety and tolerability in an initial phase 1 clinical trial and in a further phase 1b trial of multiple ascending-doses with a reduction in free serum α-syn observed (182). Phase 2 clinical trials are currently ongoing (NCT6868). Furthermore, BIIB054 another IgG 1 monoclonal antibody targeting α-syn has concluded phase 1 clinical trials (183) and is currently undergoing phase 2 (NCT03318523). However, these therapies involve regular injection with monoclonal antibodies to maintain an immune response, otherwise known as passive immunisation, another therapeutic avenue is the use of vaccines, or active immunisation in the treatment of PD. PD01A is an anti-α-syn vaccine, which maintains a higher affinity for aggregated rather than monomeric forms of α-syn. The results for the phase 1 clinical trial of this vaccine in PD patients demonstrated that it is safe and well-tolerated among treatment groups (184). Although these two strategies involve the use of antibodies to target the specified antigen, vaccination brings with it the added benefit of long-term immunisation however, passive immunisation enables treatment to cease if adverse side effects occur.

A phase 2 clinical trial is currently ongoing which investigates the use of the immunosuppressant drug, Azathioprine for treatment of PD. Azathioprine is an FDA approved, purine analogue and is used to treat numerous diseases, including multiple sclerosis. It acts by reducing B and T cell proliferation via nucleic acid synthesis inhibition (185). However, an important consideration for this therapy is the fact that it is an immunosuppressant, which exposes the patient to increased risk of infection and further illness. Another immunomodulatory therapy, Sargramostim, is an FDA approved GM-CSF that stimulates myeloid cell production and induces Treg cells. The phase 1 clinical trial demonstrated increased numbers and functionality of Treg cells while maintaining levels of T effector cells (186). Potentially enabling increased regulation of the immune response in PD.

As mentioned above, the gut-brain axis is being increasingly researched and studies have demonstrated a potential role for this pathway in PD. Importantly, gut microbiome alterations have been observed in PD patients (187) and faecal microbiome transplantation has been investigated as a therapeutic intervention for PD. A case report (188) and a preliminary study (187) have demonstrated potential benefits for this as a therapeutic strategy in the treatment of PD. Additionally, a clinical trial (NCT03808389) is ongoing in hopes that restoration of microbiome homeostasis will improve symptoms in PD patients.

These trials represent many varied and promising approaches targeting the immune system as a means of PD therapy which together may lead to future disease-modifying therapeutic strategies for the treatment of PD.

## Future Directions to Increase Our Understanding of Neuro-Immune Cross Talk

The crosstalk between the brain and the immune system in PD is clearly complex with many questions remaining to be answered. Some of these many interesting questions include the following: (1) Is the increase or reduction of various T cell subsets in the blood of PD patients reflective of the situation in the CNS, or could for example a reduction in the blood vs. healthy controls merely indicate reciprocal trafficking to the CNS? (2) What role might the different PD associated genetic mutations have on crosstalk between glial and immune cells and how that crosstalk affects dopamine neuronal survival? (3) Is the peripheral activation of α-syn reactive T cells a primary event, or is it secondary to the aggregation/alteration/mutation of α-syn or other PD associated proteins in the CNS which are then released into the periphery to activate antigen specific naïve T cells? (4) Does T cell involvement only occur in a subset of PD patients who are genetically predisposed to T cell involvement as a result of their MHC haplotype, and are certain mutated peptides preferentially presented by particular MHC haplotypes? (5) What role might PD associated genetic mutations have on mitochondrial antigen presentation and could carriers of *PINK1* or *PARK2* mutations present with an autoimmune type of PD? (6) How might all of the queries above be influenced by PD medications?

The development of new research tools in recent years, including animal and various stem cell models, has already allowed a better understanding of astrocyte and microglial function and as these methods continue to improve, we can probe more deeply into how these cells communicate with one another and also begin to answer the questions posed above. Such tools include rapid and more cost-effective sequencing at single cells resolution at various stages of the progression of pathology (189). Others that have also become available are iPSC-derived neurons, astrocytes, and microglia (190), cerebral organoids (191), cerebral organoids containing microglia (192), CRISPR screening, and high content imaging (193). The major advantage offered by iPSC is that they allow the study of the known PD associated genetic mutations where genes are expressed at native levels without any forced genetic manipulation. To gain a better understanding of the mechanisms involved in glial and immune cell crosstalk will require a deeper knowledge of the proteome of T cell subsets, astrocytes and microglia and integration with multi-omic datasets. Research will also need to focus on functional studies to gain a better understanding of dysfunctional pathways (189) that may allow development of novel therapeutic targets and the continuing development of animal models will be critical to development of our increased understanding in this area. Many exciting discoveries have been made in this field in recent years; more are sure to follow.

## Author Contributions

All authors listed have made a substantial, direct and intellectual contribution to the work, and approved it for publication.

## Conflict of Interest

The authors declare that the research was conducted in the absence of any commercial or financial relationships that could be construed as a potential conflict of interest.
